# Heterojunctions of rGO/Metal Oxide Nanocomposites as Promising Gas-Sensing Materials—A Review

**DOI:** 10.3390/nano12132278

**Published:** 2022-07-01

**Authors:** Mohd Nurazzi Norizan, Norli Abdullah, Norhana Abdul Halim, Siti Zulaikha Ngah Demon, Imran Syakir Mohamad

**Affiliations:** 1 Centre for Defence Foundation Studies, National Defence University of Malaysia, Kem Sungai Besi, Kuala Lumpur 57000, Malaysia; mohd.nurazzi@gmail.com (M.N.N.); norhana@upnm.edu.my (N.A.H.); zulaikha@upnm.edu.my (S.Z.N.D.); 2 Faculty of Mechanical Engineering, Universiti Teknikal Malaysia Melaka, Hang Tuah Jaya, Durian Tunggal, Melaka 76100, Malaysia; imran@utem.edu.my

**Keywords:** graphene, gas sensor, heterojunction, metal oxide, nanocomposite, rGO

## Abstract

Monitoring environmental hazards and pollution control is vital for the detection of harmful toxic gases from industrial activities and natural processes in the environment, such as nitrogen dioxide (NO_2_), ammonia (NH_3_), hydrogen (H_2_), hydrogen sulfide (H_2_S), carbon dioxide (CO_2_), and sulfur dioxide (SO_2_). This is to ensure the preservation of public health and promote workplace safety. Graphene and its derivatives, especially reduced graphene oxide (rGO), have been designated as ideal materials in gas-sensing devices as their electronic properties highly influence the potential to adsorb specified toxic gas molecules. Despite its exceptional sensitivity at low gas concentrations, the sensor selectivity of pristine graphene is relatively weak, which limits its utility in many practical gas sensor applications. In view of this, the hybridization technique through heterojunction configurations of rGO with metal oxides has been explored, which showed promising improvement and a synergistic effect on the gas-sensing capacity, particularly at room temperature sensitivity and selectivity, even at low concentrations of the target gas. The unique features of graphene as a preferential gas sensor material are first highlighted, followed by a brief discussion on the basic working mechanism, fabrication, and performance of hybridized rGO/metal oxide-based gas sensors for various toxic gases, including NO_2_, NH_3_, H_2_, H_2_S, CO_2_, and SO_2_. The challenges and prospects of the graphene/metal oxide-based based gas sensors are presented at the end of the review.

## 1. Introduction

Since the 1970s, the research community has achieved commendable progress on the development of chemical gas-sensing systems. The first chemiresistive gas sensor using Tin (IV) oxide (SnO_2_) was fabricated and patented by Taguchi [1]. Thereafter, ongoing research has continued to provide a better understanding of the sensing systems, such as reproducibility, selectivity, sensitivity, better stability at low-temperature operation, and fast response time [2]. In contrast, the available semiconductor materials are predominantly wide bandgap semiconductors and mostly require higher operating temperatures or Ultraviolet (UV) light to excite the valence band electrons, which limits their applications [3]. It is well-established that a significant volume of NO_2_, CO, and NH_3_ is released into the atmosphere each year via open burning, discharge from industrial wastes, and vehicle combustion. Therefore, the detection of these gases has attracted the interest of many parties due to its severe toxicity to the respiratory system of plants, humans, and animals [4,5]. Similarly, NH_3_ is a standard industrial gas that is considered toxic due to its irritant and corrosive characteristics. Even at low concentrations, the inhalation of such gases can cause serious coughing and inflammation of the respiratory tract. Additionally, direct skin and eye contact with low concentrations of NH_3_ in the air and surroundings may induce severe irritation. As a result, exposure to concentrated NH_3_ solutions, for example, those found in industrial cleaners, would result in acute corrosive injuries, such as skin burn, permanent eye damage, or blindness [6]. Toxic concentrations of these compounds are expressed in comparison to CO, which has an Acute Exposure Guideline Level-3 (AEGL-3) or lethal inhalation concentration value of 1900 mg/m^3^ over a 10-min exposure. For instance, the corresponding value for sarin gas is 0.38 mg/m^3^, which is 5000 times lower than that of CO [7]. Therefore, a prompt and effective technique to detect toxic chemical gases could protect the health and welfare of industrial personnel and those in close proximity from the aforementioned dangers.

Besides that, the most crucial concern regarding toxic gas detection is the organophosphate (OPs) compounds that are mainly used as pesticides, such as malathion, and as nerve agents in Chemical Warfare Agent (CWA) weapons. Nerve agents, including the G-type series, such as sarin, tabun, and soman, and the V-type series, such as EA-3148, VE, and VX, are hazardous due to their high volatility at low temperatures, high toxicity, and colorless and odorless physical appearance [8]. The severe symptoms include seizures, slow pulse, breathing difficulty, and coma, eventually leading to sudden death. Sarin gas is one of the noticeable examples of OPs that causes death by suffocation within 1–10 min of exposure at concentrations above 60 ppb (parts per billion) [9].

Most of the common commercial gas sensors are based on metal oxide semiconductor and polymer materials, while the fundamental sensing principles used for sensing are the optical gas sensor method [10], the calorimetric gas sensor method [11], the electrochemical gas sensor method [12], the capacitance gas sensor method [13], the acoustic wave gas sensor method [14], and the chemiresistor gas sensor method [15]. Nevertheless, various factors can limit the effectiveness of these gas sensors, such as operational cost, difficulty in achieving high sensitivity level (up to ppb), poor selectivity, limited life span, poor repeatability, complex miniaturization, and high power consumption [16]. Other gas sensors that demonstrate high sensitivity, reliability, and precision include Liquid Chromatography (LC) [17], Gas Chromatography-Mass Spectrometry (GCMS) [18], Ion Chromatography (IC) [19], Atomic Emission Detection (AED) [20], Capillary Electrophoresis (CE) [21], CE coupled with flame photometry [17], and CE with conductivity detection [22]. All these advanced gas-sensing technologies require optimum performance, expensive equipment, and complex procedures by highly trained personnel, as well as being tedious, and incompatible with on-field analysis.

Therefore, it is necessary to highlight the pros and cons of each type of gas sensor depending on certain operating and environmental conditions as well as the production cost, which varies among the method classes. Each sensor technology is continuously developed to achieve an optimum performance, and thus the trend is to fabricate miniaturized sensors with ideal properties. An ideal sensing system is defined by several variables, which consist of sensitivity, selectivity, high response time, and fast recovery time. In view of this, microfabrication techniques have recently been utilized to fabricate miniaturized sensors that offer small-sized, low cost, low power consumption at low operating temperatures, and other features that make them an ideal sensor. Table 1 shows the performance comparison between the graphene-based gas sensor and other types of gas sensors.

In recent years, chemiresistor technology has been developed for various applications due to its ability to provide accurate real-time data via miniaturized devices that consume a small amount of electricity [23]. The sensing performance of chemiresistive materials depends precisely on their microstructure. As previously stated, these properties influence the sensing performance by multiplying the number of active sites available for the detection of analytes. To synergize the chemical gas detection technique, nanomaterial-based gas-sensing materials have gained substantial interest due to their high surface-to-volume ratio, fast response and recovery time, high sensitivity, selectivity, reversibility, and stability, as well as promising electrical, optical, and thermal performances. Different carbon materials, ranging from CNTs, charcoal, graphene, graphene oxide (GO), and reduced graphene oxide (rGO), are renowned as useful gas sensors due to the simplicity in which their sensitivity can be tailored using simple chemical modifications [24,25]. Referring to Chava et al. (2019), the tailored structures of nanomaterials, nano size and dimensions have established a great future for the use in the gas-sensing layers, and the advantages of these nanostructured materials include a high specific surface area, a large surface-to-volume ratio, and more active sites on the surface. Since the nanostructured materials have a large surface-to volume ratio, a larger number of surface atoms will be available on the surface and there will be more interactions between gas molecules and the material which is the key factor controlling the sensor performance. Thus, nanostructured sensing layer materials can show higher performances when compared to the bulk counterparts [26].

Despite the promising advantages of graphene, the lack of surface functionalities in pristine graphene limits its applicability as a gas sensor. Regarding this matter, hybridization with metal oxides is still being investigated for sensitive and fast response detection. Metal oxides are highly preferable given their high surface-to-volume ratio, high porosity, and easily flexible characteristics, such as composition, structure, morphology, and diameter [27]. George et al. (2018) stated that a metal oxide was an ideal material to enhance the performance of graphene-based sensors [28]. Examples of metal oxide nanoparticles that have been utilized as electrochemical sensors include titanium dioxide (TiO_2_) [29], zinc oxide (ZnO) [30], cerium oxide (CeO_2_) [31], iron oxide (Fe_2_O_3_) [32], nickel oxide (NiO) [33], tin dioxide (SnO_2_) [34], cobalt (II, III) oxide (Co_3_O_4_) [35], and indium (III) oxide (In_2_O_3_) [36,37,38]. Metal oxides offer multiple benefits, which comprise the ability to expedite the development of a three-dimensional (3D) conductive porous network to enhance the charge transfer pathway and electrical performance as well as the ability to modify the surface properties of materials [39,40]. Furthermore, metal oxide-based gas sensors have also been used in the past decades to detect analytes at high working temperatures that are sufficient enough to induce a gas interaction with oxygen ionosorbed over the semiconductor, resulting in a change in the material resistance. For that reason, the detection of highly explosive gases at high working temperatures may increase the risk of fuel ignition. Therefore, toxic gas detection at room temperature is critical to avoid unexpected explosions when hydrogen is mixed with atmospheric oxygen at a Low Explosive Limit (LEL) concentration of 4% [41]. In example, In_2_O_3_ is a typical *n*-type metal oxide semiconductor, and it is widely utilized as a gas-sensing layer for the detection of variety of gases. The principle behind the working of In_2_O_3_ and any other metal oxide resistive gas sensor is associated with the variation of surface resistance as a function of the surrounding temperature and gas atmosphere. The adsorbed gas molecules on a metal oxide semiconductor surface lead to redox reactions by serving as electron donors or acceptors which is estimated by the analyte’s reductive or oxidative nature. The changes in the resistance of materials occurs due to the gas–solid interface reactions by altering the charge carrier concentration near the surface of the semiconductor.

Since the gas-sensing mechanism is closely related to the tailored morphology, nano particle size, and large surface-to volume ratio of nanostructured materials, numerous nanostructured metal oxide materials with various morphologies and architectures with their gas-sensing properties have been reported. Among these, hierarchical structures derived from low dimensional nano-building blocks, such as nanorods, nanoparticles, and nanoflakes, are very worthy of in-depth study. These hierarchical structures could have the characteristics of less agglomerated states, good surface permeability, effective charge transport, and gas diffusion which can enhance the gas-sensing properties [42].

Furthermore, the synergistic effects of graphene hybridization with metal oxides revealed high levels of electrochemical activities, assisting in improving the electrochemical sensor selectivity and sensitivity [43]. The formation of *p*-*n* heterojunction nanocomposites could modify and design the gas-sensing capabilities of gas sensors by changing the electrical performance near the hetero-interfaces. Besides, the high specific surface area of nanocomposites would provide more active sites available and would promote gas adsorption for the specific oxygen adsorption to project a high responsiveness gas sensor. In addition, several rGO/SnO_2_-based gas sensors have been reported to exhibit a high sensing performance [44]. As compared to pristine metal oxide, such as SnO_2_, the rGO/SnO_2_ nanocomposites overcome the limits of the high operating temperature of the pristine SnO_2_ semiconductor material and can decrease the operating temperature to 50 °C, even as low as room temperature range. Additionally, the hybridization of rGO with SnO_2_ can effectively prevent the agglomeration of SnO_2_ nanoparticles and reduce the SnO_2_ grain size [45]. As compared to pristine SnO_2_ semiconductor materials, the resistance of rGO/SnO_2_ can also be reduced by several orders of magnitude [46]. A study was conducted that hybridized the In_2_O_3_ metal oxide with gold (Au) nanoparticles in order to form a core–shell hybrid hetero nanostructures-based gas sensor for H_2_ detection. The Au/In_2_O_3_ core–shell nanoparticles showed a greater sensitivity and selectivity towards H_2_ gas, with the highest response of 34.38 at 100 ppm of gas level, whereas the pristine In_2_O_3_ showed a response of only 9.26. However, the effective operating temperature was achieved at 300 °C [47].

**Figure 1:**
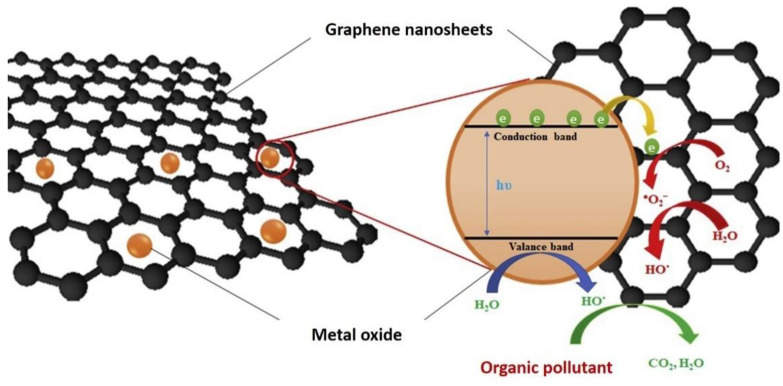
Schematic diagram of the hybrid graphene/metal oxide nanocomposite for toxic gas detection. Reproduced from ref. [43].

**Figure 2:**
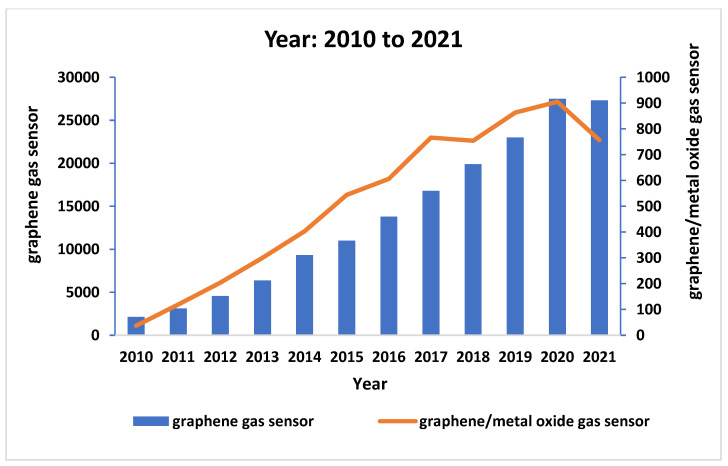
Research progress on the development of graphene/metal oxide as a chemical sensor from 2010 to 2021.

Therefore, this review emphasizes the significance of rGO hybridization with metal oxide as gas sensors for the detection of various toxic gases, such as NO_2_, NH_3_, H_2_, H_2_S, CO_2_, and SO_2_. The basic working mechanism and their performance as gas sensors are briefly discussed in this review. For this review, the research progress encompasses the development of the hybridization of graphene with a metal oxide as a gas sensor (specific keyword: “graphene gas sensor” and “graphene/metal oxide as a gas sensor”) from the year 2010 to 2021, which was obtained from Google Scholar on 5 October 2021. The keyword “graphene/metal oxide as a gas sensor” linked approximately 6257 publications in Google Scholar with an increasing trend. This indicates that the keyword “graphene/metal oxide as a gas sensor” has become an intriguing subject to explore and develop, which will eventually benefit public health and safety. The manuscript ends with a concise conclusion and future projections of graphene/metal oxide nanomaterials as potential gas sensors. Figure 1 shows the schematic diagram of the hybrid structure of graphene/metal oxide nanocomposites, while Figure 2 illustrates the increasing research trend related to the development of graphene/metal oxide as a gas sensor.

## 2. Unique Characteristics of Graphene as a Gas Sensor

In 2004, Geim and Novoselov [48] led a pioneering study to isolate a new type of material that was made up of carbon atoms containing an sp^2^ bonded into a honeycomb lattice configuration with a 0.142 nm distance between them called graphene [49]. Other carbon allotropes had a basic structure that was composed of arrays of individual graphene layers bound together by Van der Waals interactions. The unique graphene characteristics are as follows:

(1) High strength, which is attributed to the covalent bonding within the layers, and weak lateral bonding, allowing it to easily slide and slip;
(2) The thinness of the graphene atomic formation has a relatively high absorbance of 2.3% based on the fine-structure constant [49] and a high thermal stability, e.g., when annealed up to 250 °C;
(3) The high in-plane thermal conductivity up to approximately 5 × 10^3^ W/mK provides an excellent heat dissipation and thermal interface [50,51];
(4) The electronic structure with a zero bandgap within contributes to its high electron mobility (10^5^ cm^2^/Vs) [46], which is much higher than that of other electronic nanomaterials, such as CNTs (1.096 × 10^4^ cm^2^/Vs) [52,53] and certain conducting polymers, for example, poly(3-hexylthiophene) (P3HT) (10^−1^ cm^2^/Vs) [54] and polyaniline (PANI) (0.69 cm^2^/Vs) [55];
(5) High carrier density (10^13^/cm) [56], room temperature Hall effect [57], and low electrical noise of approximately 1–100 kHz, which is similar to other metals and semiconductors [58,59];
(6) As reported by Li et al., (2019), the developed rGO wrapped sponge for highly efficient oil/water separation exhibited incredible mechanical strength, extremely high flexibility, bendability, and compressibility of up to 100 squeezing cycles [60]. Nonetheless, the hexagonal arrangement showed that graphene and CNTs have a relatively high elasticity with a Young’s modulus of 1 TPa, a third-order elastic stiffness around 2 TPa, and a shear modulus of approximately 80 GPa [61,62].

**Figure 3:**
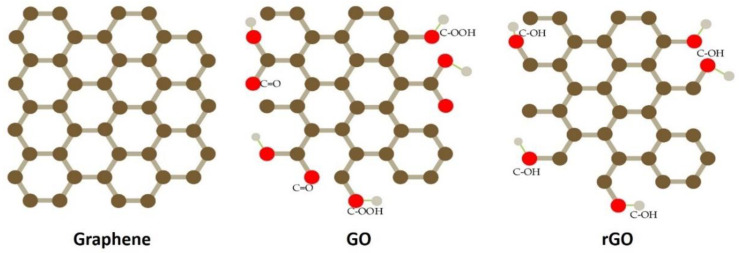
The schematic diagram of the structure of graphene, GO, and rGO. Reproduced from ref. [64].

Pristine graphene, GO, rGO (Figure 3), and its derivatives have been demonstrated to show impressive sensing behaviors, including a high carrier mobility at room temperature, low electrical noise, and a unique 2D honeycomb lattice with a large theoretical surface area of 2630 m^2^/g [50]. Unlike one-dimensional (1D) materials, such as CNTs, 2D-structured materials offer better screen charge fluctuations [51,63]. The fact that the electronic characteristics of graphene are substantially influenced by gas molecule adsorption is possibly the most substantial reason for it being promoted as a viable gas-sensing material. The planar structure of graphene facilitates the fabrication of Hall patterns and four-probe testing, reducing contact resistance implication, and permitting researchers to focus solely on the active sites [52]. The characteristics of graphene as a gas sensor and their remarks are listed in Table 2 [54,55,56,57,58,59].

According to Novoselov et al. (2007), the first graphene-based gas sensor revealed that the adsorbed molecules changed the local carrier concentration in graphene with each electron, resulting in resistance changes. Consequently, the magnitude of the resistivity changes determines the type of gas being induced, whether an electron acceptor (such as NO_2_, H_2_O, iodine) or an electron donor (such as NH_3_, CO, ethanol) [61,62]. Graphene sheets and gas analytes may interact through various pathways, from mild Van der Waals forces to strong covalent interactions, and the reactions that change the electronic structure of graphene could be easily observed using simple electronic methods.

There are a few issues to consider when graphene is employed as a gas sensor:

(1) It is not mass-producible;
(2) Functional groups are not required for gas and chemical vapor adsorption;
(3) It has a zero bandgap.

For this reason, graphene is considered a metallic-behaving material. In fact, it is more metallic than conventional metals. This is only true when the size of the graphene layer is within several micrometers or even hundreds of nanometers. However, a different bandgap could be created when the dimension of graphene is reduced to narrow ribbons with a width of 1–2 nm, which could produce semiconducting graphene with a potential application in transistors [65]. Additionally, Guo et al. (2018) stated that sheet stacking lowered the surface area of graphene, thereby paving the way for the advancement of graphene-based gas sensors [66].

Graphene can be oxidized to form GO, which contains hydroxylated functional groups comprising carboxyl (-COOH), hydroxyl (-OH), epoxy (C-O-C), carbonyl (-C-OH), ketone (-C=O), and 5-membered and 6-membered ring lactols (O-C-O). Therefore, GO differs from pristine graphene in terms of the physical and chemical characteristics, making GO particularly useful for gas sensing. In comparison to pristine graphene, the attached hydroxylated functional groups on GO increase its hydrophilic nature and also provide limitless surface functionalization opportunities. This intrinsically increases the sensitivity of GO to water molecules, making it a potential humidity sensor [67].

**Figure 4:**
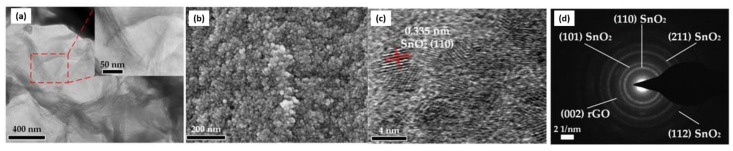
(**a**) TEM image of rGO with the inset HRTEM image of rGO at 50 nm, (**b**) SEM image of SnO_2_, (**c**) HRTEM image of rGO/SnO_2_ prepared via the hydrothermal process at 180 °C and reaction time of 12 h, and (**d**) Selected Area Electron Diffraction (SAED) pattern of an rGO/SnO_2_ nanocomposite. Reproduced from ref. [74].

Since rGO is functionalized through the reduction of GO that contains specific functional groups and defective sites, rGO can be fabricated through an easy and economical approach for large-scale applications, especially as a gas sensor. For example, Lu et al. (2011) demonstrated the fast response and recovery of NH_3_ detection using rGO under a positive gate potential (*n*-type conductance), which showed better characteristics in the *p*-mode at zero or negative gate [68]. Following the recent findings, numerous rGO-based gas sensors and their derivatives have been established [67,69,70]. For instance, a NO_2_ gas sensor was developed by sandwiching an rGO micro-sheet between two Au electrodes [71,72,73] The electron transfers from rGO to the adsorbed NO_2_ molecules enhanced the conductance of the *p*-type rGO sheet, leading to an improvement in the sensing capacity. Figure 4 shows the image of rGO/SnO_2_ layers viewed under Field Emission Scanning Electron Microscopy (FESEM), Transmission Electron Microscopy (TEM), and High-Resolution Transmission Electron Microscopy (HRTEM).

## 3. Synthetization Methods for the Fabrication of rGO

The three major steps to synthesize rGO comprise oxidation or intercalation, exfoliation, and reduction. The 2D monolayer of a sp^2^-hybridized honeycomb-like carbon matrix graphene has an excellent performance in terms of electronic conductivity, specific surface area, porosity, thermal stability, and mechanical strength. During the preparation of graphene-supported semiconductor nanocrystals, graphene acts as a surfactant to avoid the agglomeration of metal oxides as well as an oxidant to oxidize metal ions with low oxidation states. Since it is an excellent conductive material, hybridization with graphene can enhance the electron transfer to the target gas (or interior of nanocomposites) and improve gas diffusion, especially for heterojunction nanocomposites with interconnected porous structures and high specific surface areas [75].

In order to fabricate materials that exhibit comparable properties to that of pristine graphene, extensive studies have been carried out to remove the oxygen functional groups in GO [76]. This reduction can be achieved via several approaches, ranging from thermal to chemical to electrochemical techniques, each of which leads to differences in the morphology, electrical properties, and other performances of the fabricated material. The key development factors in GO reduction include the carbon-to-oxygen (C/O) ratio of the end product, selectivity in removing a single type of oxygen group (hydroxyl vs. carboxyl vs. epoxy), healing of the surface defects of the GO from oxidation, and the selection of green-reducing agents, as well as maintaining or improving the desired properties (mechanical strength, conductivity, optical properties, solubility/dispersibility of nanosheets) of the GO. Zang et al. (2018) stated that the commonly used chemical reducing agents for the reduction process, such as hydrazine and hydroiodic acid (HI), are highly toxic [77], in addition to metal hydrides or hydrohalic acids that have a corrosivity and toxicity effect [78]. Besides, the chemical reduction of graphite oxide colloidal suspensions has been considered an effective route to synthesize graphene sheets due to their simplicity, reliability, the capability for large-scale production, and exceptionally low cost [79].

Nevertheless, such chemically reduced products suffer from a low C/O ratio [78] and the quality of the structure of the graphene sheets in these rGO is very poor compared to the graphene produced via mechanical/physical exfoliation or Chemical Vapor Deposition (CVD). Additionally, the reduction reaction employed in aqueous suspensions caused an agglomeration of the rGO sheets. For the preparation of high-quality graphene, it was revealed that high-temperature annealing from the thermal reduction method is necessary, and the layers of rGO are expanded through the decomposition of oxygen groups into CO and CO_2_ gases at elevated temperatures [80]. The annealing is usually carried out under vacuum conditions [81], or in an inert [82] or reduced atmosphere [83]. The thermal treatments result in a much higher degree of reduction than that of the conventional chemical reduction treatments, as the sp^2^ carbon domains are restored and the electrical properties of GO nanosheets are improved. In contrast to the inert atmosphere, the annealing process under the reducing atmosphere facilitates the formation of Highly Reduced Graphene Oxide (HRGO). Besides, the heating temperature during the thermal exfoliation of GO dictates the removal of oxygen functional groups from the GO surface, consequently affecting the reduction effectiveness of GO. The increase in annealing temperature from 1000 °C to 2500 °C results in an improved crystallization of the graphene product, particularly the enhanced electrical conductivity up to 550 S/cm with the formation of highly crystalline rGO aerogel [84]. Furthermore, the annealing reduction of GO under the NH_3_ condition leads to the formation of N-doped graphene [85].

One of the early methods to reduce GO for the formation of rGO is through the use of UV light in the presence of a TiO_2_ catalyst [86]. Electrochemical reduction is also possible and does not require the use of chemical agents in which the reduction is exclusively driven by electron exchange between GO and the electrodes of a typical electrochemical cell [87]. In recent years, several reports have shown the effective use of green reducing agents, such as ascorbic acid, sugars, amino acids, and even microorganisms through microbial degradation processes [88] to synthesize rGO [76]. Interestingly, various studies have explored the synthesis of nanomaterials through the utilization of bacteria to reduce metallic salts since bacterial cells have an extremely diverse metabolic pathway. Depending on the species, certain bacterial cells have the capacity to directly or indirectly hydrolyze acid groups associated with nanosheets of carbon, especially oxygen atoms. This opens the opportunity for bacterial cells to synthesize nanomaterials, including GO, under a specific reduction mechanism. GO oxide can act as a final electron acceptor to capture the electron coming from the bacterial respiration process to produce GO [89].

The microwave heating process, which is considered a promising technique with low energy consumption, is widely used in the preparation and modification of carbonaceous materials [90]. Several studies have also adopted the microwave-irradiation method for the large-scale production of high-quality rGO from GO [91,92]. Relative to the large consumption of energy in high-temperature annealing above 1000 °C, microwave-assisted heating is an efficient method for the expansion and reduction of precursors prepared via the modified Hummers method into microwave-exfoliated GO [93]. The microwave-assisted heating significantly enhances the energy transfer directly to the reactants, which causes an instantaneous rise in internal temperature, which makes this process a fast and energy-saving technique. In several studies, the microwave-assisted reduction of GO was performed in aqueous or organic media [94,95]. For example, Chen et al. reported a microwave hydrothermal reduction of GO in a mixed solution of *N*, *N*-dimethylacetamide, and water (DMAc/H_2_O) (0.56 mg/mL). The microwave irradiation was set to 800 W and the rGO achieved a conductivity of 200 S/m which was accomplished within 10 min [96].

## 4. Metal Oxide as a Gas Sensor

**Figure 5:**
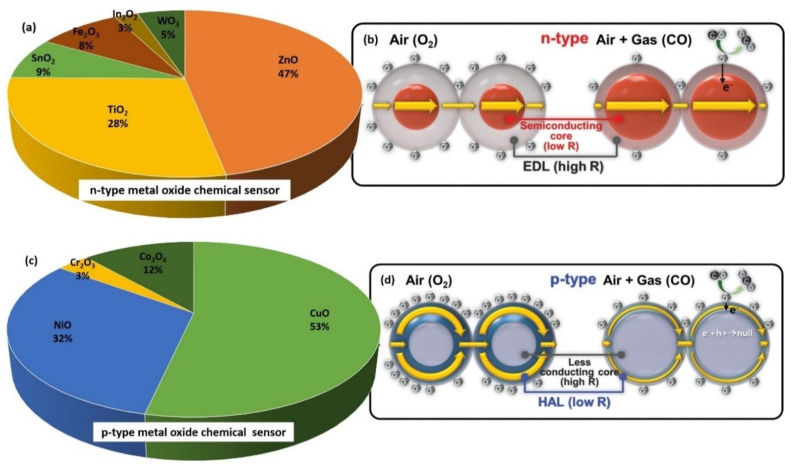
Search study on (**a**) *n*-type and (**c**) *p*-type metal oxides as gas sensors from 2020 to 2021, (**b**,**d**) gas-sensing mechanisms of *n*-type and *p*-type metal oxide chemical sensors. Reproduced from ref. [97].

The search results from Google Scholar between 2020 and 2021 (search date: 22 June 2021) regarding both *n*-type and *p*-type metal oxides as sensing materials for gas sensor applications are shown in Figure 5. The keywords used for the search included “chemical formula” and “chemical sensor” of the metal oxide, for example, “SnO_2_ chemical sensor” was used to search for SnO_2_ chemical sensors. The “ZnO chemical sensor” search for an *n*-type metal oxide, which is considered less toxic, cheap, and easy to handle compared to other metal oxides, recorded the highest result with 41,700 articles, which was equivalent to 47%. On the contrary, the “CuO chemical sensor” search for *p*-type metal oxide registered 17,300 articles, which was equivalent to 53%. In total, the *n*-type metal oxide has received greater attention as a chemical sensor and one of the reasons for the declining interest in *p*-type metal oxide chemiresistors is their low gas response [97].

Metal oxide nanoparticles exhibit a variety of electrical and photochemical properties due to their nanosize, high stability, and high surface area [28]. The primary functions of metal oxides in gas sensor devices include the ability to reinforce the conductive sensing interface, catalyze the expansion of nanoparticles with metals, electrically connect redox centers in proteins (biosensors) to the surface of the transducers, and increase the speed of detection and sensor responsiveness [98]. The rapid electron transfers between the transducer and analyte molecules are considered “electronic wires” and “electrocatalysts” given the nanoscale and structure of metal nanoparticles, [99]. The high affinity of metal oxides allows the working electrode surface to be developed using various methods, including physical adsorption, electrodeposition, chemical covalent bonding, and electropolymerization [100]. However, certain limitations of metal oxides have been reported, including a large bandgap caused by their function as semiconductors or even insulators, poor ion transport kinetics [101], and electrode film pulverization caused by the pronounced volume expansion and contraction during the charging or discharging process [102]. These constraints may be overcome through metal oxide hybridization with carbonaceous materials, such as graphene and CNTs, as well as with other metal nanoparticles and conductive polymers [25]. Apart from that, efforts to effectively increase the gas-sensing characteristics of metal oxides have concentrated on surface modification [103], doping [104], and morphological or nanosize modifications [105].

Changes in conduction routes in *n*-type and *p*-type metal oxide semiconductor sensors are responsible for the observed differences in gas responses. At a temperature range of 100–450 °C, oxygen adsorption and ionization to O_2_^−^, O^−^, and O^2−^ establish the formation of the electronic core (semiconducting)-shell (resistive Electron Depletion Layer (EDL)) in *n*-type metal oxide semiconductors (Figure 5a,b). Conduction necessitates the electron to pass the Schottky barrier back-to-back at the inter-particle interaction when the particle size exceeds twice the thickness of the EDL (grain boundary). Therefore, the serial connection between the semiconducting cores and resistive shells explains the conduction phenomenon. On the other hand, the ionized oxygen (O_2_^−^, O^−^, and O^2−^) on the surface of *p*-type metal oxide semiconductors attracts the opposite charge of the majority charge carriers (holes), forming distinct electronic core (less conducting)–shell (semiconducting Hole Accumulation Layer (HAL)) structures (Figure 5c,d). Once the particle diameter twice exceeds the diameter of the HAL, the conduction is determined through the simultaneous competition of two causes: (1) along with the semiconducting HAL with the smaller cross-sectional area and (2) along with the less conducting core with the larger cross-sectional area.

When the electrons are injected into the sensing materials by the reaction between ionized oxygen and the reducing gas, the electron concentration in the EDL increases in *n*-type chemiresistors, whereas the hole concentration in the HAL decreases in *p*-type chemiresistors, leading to the opposite chemiresistive variations [97]. According to Jeong et al. (2020), *n*-type metal oxide semiconductors with serial conduction paths were more beneficial than *p*-type semiconductors with parallel conduction paths and achieved a greater variation in the overall sensor resistance.

Kim and Lee (2014) highlighted three noticeable differences in chemical-sensing features for *n*-type and *p*-type metal oxide-based gas sensors based on the receptor function, conduction route, and gas-sensing method with differing main charge carriers:

(1) The formation of an EDL in *n*-type metal oxide semiconductors or a HAL in *p*-type metal oxide semiconductors is due to the oxygen adsorption with a negative charge;
(2) The conduction in *n*-type metal oxide semiconductors occurred via serial paths (such as semiconducting particle cores and resistive interparticle contacts), whereas in *p*-type metal oxide semiconductors, the conduction occurred via parallel paths (such as resistive particle cores and semiconducting near-surface regions);
(3) A chemiresistive dissimilarity was detected at the interparticle contacts in *n*-type metal oxide semiconductors, whereby the chemiresistive variation occurred at the near-surface regions in *p*-type metal oxide semiconductors [106].

Kim and Lee (2014) also mentioned that the formation of a *p*-*n* heterojunction by the metal oxide semiconductor materials could modify and design the gas-sensing capabilities of gas sensors by changing the electrical performance near the hetero-interfaces. Additionally, the specific oxygen adsorption of *p*-type metal oxide could be utilized to project a highly responsive gas sensor. As a result of these opposing characteristics, the response of metal oxide-based gas sensors is defined in terms of the active layer resistivity, which is defined differently depending on the type of measurement.

In the presence of reducing analytes, such as CO, NH_3_, and ethanol, the resistance ratio (Rg) is expressed as (Ra/Rg), while the response of an *n*-type material to an oxidizing analyte, such as NO, NO_2_, and O_3_, is expressed differently as (Rg/Ra) [107,108] and is conversely for *p*-type sensors [109,110]. The resistance reacts differently when *n*-type and *p*-type metal oxide sensors interact with the reducing analyte. For a *p*-type metal oxide sensor, the resistance increases when it interacts with the reducing analyte and decreases when it interacts with the oxidizing analytes [111]. In contrast, the resistance decreases when an *n*-type metal oxide sensor interacts with the reducing analyte, and the resistance increases when it interacts with the oxidizing analyte.

Meanwhile, a *p*-*n* heterojunction is made up of a number of synergistic types of sensors, where they are definitively classed into their dominating charge carrier with *n*-type or *p*-type characteristics based on the variation of resistivity in response to the change in analyte concentration. This is to establish a thorough knowledge and practical determination of *n*-type and *p*-type analyte-sensing performances [24,112]. The responsiveness (S) of a *p*-type graphene-based gas sensor is determined using Equation (1):

S (%) = (R_g_ − R_o_)/R_o_ × 100 (1)

where R_o_ and R_g_ represent the electrical resistance of graphene-based sensors before and after the exposure to the analyte (such as CO), respectively.

Chatterjee et al. (2015) reported that metal oxide nanostructures, such as ZnO, SnO_2_, WO_3_, and Cu_2_O, have been extensively explored for sensing applications primarily due to their large specific surface area, excellent mechanical flexibility, and good chemical stability [113,114,115,116]. Nevertheless, metal oxide-based gas sensors exhibit noticeable weaknesses as they require high operating temperatures (150–500 °C), leading to increased power consumption. Therefore, it would unfavorably affect the integration and long-term stability of the system [117]. Furthermore, the high working temperatures may pose a risk for on-site detection, whereby explosive and toxic gases may be present in the environment. Despite the worthy advantages, it has also been reported that metal oxide sensors have a generally poor selectivity in the detection of similar gaseous species [118].

## 5. Basic Operating Principle of Graphene/Metal Oxide-Based Gas Sensors and Their Detection Mechanisms

One of the significant advantages of the graphene-based gas sensor is the near-metallic conductivity of graphene and the possible inherent-amplified sensing configuration [119]. The high specific surface area of graphene may induce synergetic effects in achieving targeted gas response at room temperature when hybridized with metal oxides, especially on sensitivity and selectivity characteristics. Meanwhile, graphene and rGO exhibit ambipolar behavior (contains both electrons and positive ions moving in opposite directions) and almost symmetric behavior in the electron and hole doping regions. They also showed –p (hole)-dominant conducting properties due to the adsorbed water and oxygen molecules. Additionally, the configuration of the graphene sheets with an *n*-type metal oxide could lead to the formation of a *p*-*n* junction. As a result, a novel nanostructure would result in a large surface area and adsorption capacity, subsequently exhibiting high electrocatalytic properties. This is far better compared to those of the individual materials, including pristine graphene, especially for chemical sensors [120,121,122].

When *n*-type metal oxides are exposed to oxidizing gases, the electrons continue to be captured by the surface of semiconductors. This increases the width of the EDL, which increases the electrical resistance. However, when the metal oxide interface is exposed to reducing gases, the gas molecules act as electron donors to the metal oxide interface, which reduces the width of the EDL and lowers the resistance of the gas sensor. On the contrary, when *p*-type metal oxides are exposed to oxidizing gases, the width of the HAL increases as electrons are captured by the surfaces of the metal oxides, decreasing the resistance. When exposed to reducing gases, electrons are released to the metal oxides, reducing the width of the HAL, and increasing the gas sensor resistance. The working temperature affects the kinetics, conductivity, and electron mobility of the metal oxide, which then significantly influences the sensing capacity of metal oxide-based gas sensors [123,124]. Referring to Patil et al. (2016), an adequate source of thermal energy was required to overcome the potential barrier and to obtain the required electron mobility of the metal oxide-based sensors that operate above 200 °C. Therefore, the hybridization of graphene with metal oxides could improve the sensing capabilities at lower operating temperatures.

**Figure 6:**
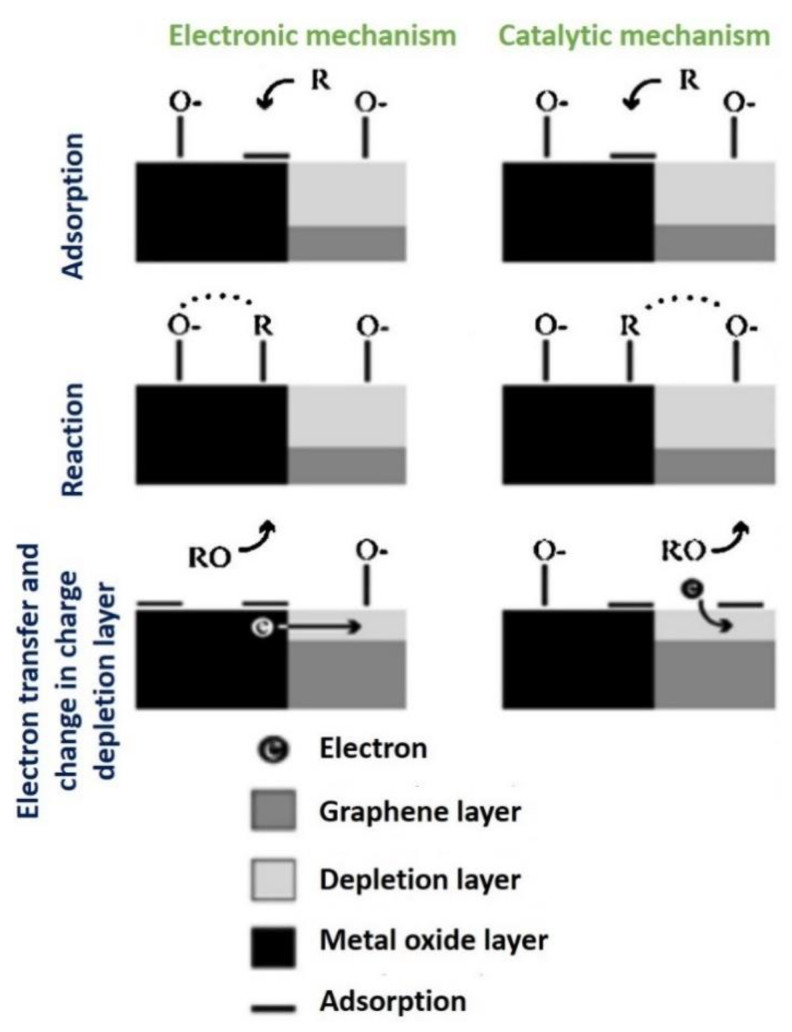
Schematic diagram of the general steps involved in the electronic and catalytic interactions between the graphene-based gas-sensing layers and the metal oxide.

Chatterjee et al. (2015) proposed two models based on (1) the oxygen ionosorption and (2) the presence of vacant oxygen in the interpretation of gas-sensing mechanisms for graphene-based gas sensors [119]. Figure 6 depicts the common steps involved in the response of graphene-based sensors when exposed to air and a reducing gas (R). Equations (2) and (3) are used to formulate Equation (5), which describes the oxygen adsorption:

O_2_ (gas) + e^−^ ⇄ O_2_^−^ (adsorption) (2)

O_2_^−^ (adsorption) + e^−^ ⇄ O_2_^2−^ (adsorption) ⇄ 2O^−^ (adsorption) (3)

Upon exposure to a reducing gas, such as CO, the occurred surface reactions follow that of Equations (4) and (5):

CO + O^−^ (adsorption) → CO_2_ + e^−^ (4)

2CO + O_2_^−^ (adsorption) → CO_2_ + e^−^ (5)

According to Jeevitha et al. (2019), other factors that influence the gas-sensing mechanism of rGO/metal oxide nanocomposites include the porosity of the nanomaterials, the specific surface area, and the formation of *p*-*n* or *p-p* heterojunction configurations. The principle of metal oxide semiconductor sensors is based on the resistance changes caused by the reaction between target gas molecules and the sensitive surface. In an rGO/WO_3_ nanocomposite, tungsten trioxide (WO_3_) acts as an *n*-type semiconductor, whereas rGO acts as a *p*-type semiconductor. The predominance of electrons and holes in *n*-type and *p*-type materials was well-established in which a depletion layer is formed at the interface when they come into contact, resulting in a *p*-*n* heterojunction. The rGO/WO_3_ sensor exhibited *p*-type behavior when NH_3_ was detected. Moreover, the rGO exhibited a higher work function and defects on the prepared nanocomposite surface, resulting in multiple adsorption sites for NH_3_. Therefore, when exposed to NH_3_, the NH_3_ molecules were adsorbed onto the nanocomposite surface, and the mechanism of the adsorbed O_2_^−^ and NH_3_ liberated free electrons and neutralized the holes in the rGO. Consequently, charge conduction channels became narrower, increasing the width of the EDL, and thus increasing the sensor resistance [125].

## 6. Transduction Mechanism for Chemiresistive-Based Sensors

In this review, the detection of toxic gases is based on chemiresistive sensors. The chemiresistive sensors are mainly based on the use of an Interdigitated Electrode (IDE), where the sensing layer is placed on top of the sensor to monitor chemical changes in the environment. When the sensor is exposed to different analyte concentrations, the interaction between the sensing layer and the analyte causes a change in the resistance. To date, traditional resistive chemical sensors are being developed further in terms of sensitivity, power consumption, response time, portability, and miniaturization. Electrical resistance is the easiest and most cost-effective electrical factor that can be measured using low power consumption. However, achieving high sensitivity and selectivity requires an extremely sensitive transduction mechanism/transducer that provides a high signal-to-noise ratio and a molecular recognition element that is extremely specific for the target analyte, respectively [126].

The transducer converts physical parameters, such as resistance, capacitance, and inductance, into electrical parameters, as well as providing a sensing voltage or current signal that can measure the magnitude and frequency. Thus, in electrically transduced chemical sensors, where gas molecules directly interact with the sensing material, these interfaces play a key role in defining the sensitivity, stability, and bio-compatibility of the sensing device with a fast response at lower concentration of toxic gases. The primary charge carrier and the type of toxic gases interacting with the active layer of the chemical sensor induce the resistance change upon the gas exposure. From a practical application, a sensor presents a rather high selectivity because a high response usually enhances the detection limit, whereas a better selectivity enables the gas sensor to exclusively respond to a certain target gas. Although several hybridized sensors make up a *p*-*n* heterojunction, the changes in resistivity when reacting with different analyte concentrations are sorted into their single dominant charge carrier trait of either *n*-type or *p*-type behavior. Table 3 summarizes the characteristics of the sensor response based on the resistance sensitivity of the *n*-type and *p*-type metal-based chemical sensors with various analytes [111]. In comparison, Table 4 displays other types of chemical sensors based on their transduction mechanism [127].

## 7. Performance of the Heterojunction Configuration of rGO/Metal Oxide-Based Gas Sensors

### 7.1. Nitrogen Dioxide (NO_2_) Gas Detection

Recently, Lee et al. (2019) developed an rGO/ZnO-based gas sensor for NO_x_ detection [142] with a good reproducibility performance via the thermal annealing process in an argon atmosphere at 300 °C for 1 h. The argon gas was used to eliminate the oxygen-containing functional groups on the nanocomposite during the annealing process. The sensing responsivity, response time, and recovery time of the rGO/ZnO sensors toward NO_2_ gas were 47.4%, 6.2 min, and 15.5 min, respectively. In contrast, the sensing responsivity, response time, and recovery time of pristine rGO gas sensors operated under 100 ppm NO_2_ environment at room temperature were 19.0%, 10.3 min, and 75.9 min, respectively. Based on the results, the significant binding energy between the NO_2_ gas and the oxygen-containing functional groups was due to the delayed recovery time of the pristine rGO towards NO_2_ gas sensors. Not only were the essential reactive electrons obtained from the oxygen vacancies in the ZnO material but the elimination of oxygen-containing functional groups and the formation of C-O-Zn bonds also contributed to the reduction in the recovery time and the improved detection sensitivity.

**Figure 7:**
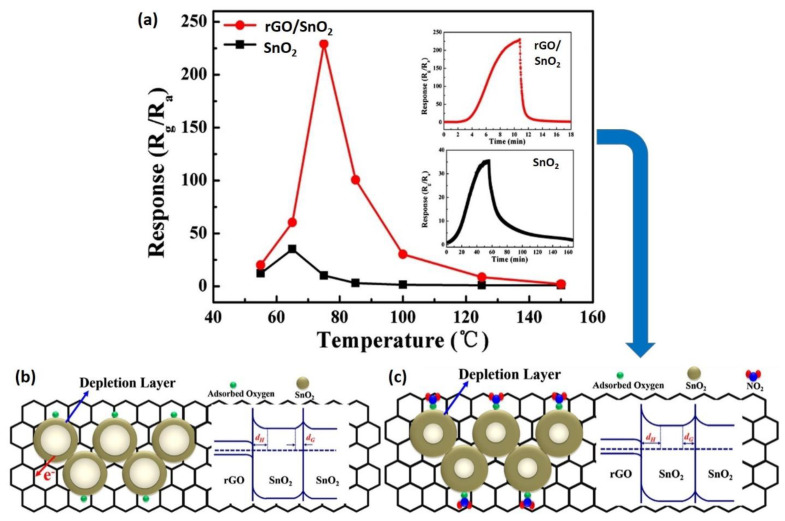
(**a**) The rGO/SnO_2_ response towards NO_2_ at 75 °C. Schematic diagram of the energy bandgap model for the sensing mechanism of the SnO_2_/rGO nanocomposite-based NO_2_ sensor under (**b**) air conditions and (**c**) NO_2_ atmosphere. Reproduced from ref. [143].

In another study, Wang et al. (2019) characterized the hybridized rGO with SnO_2_ metal oxide (rGO/SnO_2_) as an ultrasensitive NO_2_ sensor [143]. The sensor was synthesized via a combination of hydrothermal and chemical solution deposition techniques. The presence of SnO_2_ nanoparticles on the surface of rGO was multiplied to aid the transfer process, which displayed an excellent rGO/SnO_2_ performance and enabled the detection of NO_2_ gas at room temperature at a concentration of 1 ppm [144]. Additionally, the SnO_2_ nanoparticles with an average diameter of 3–5 nm contained abundant oxygen vacancies, facilitating analyte adsorption by increasing the number of donor SnO_2_-x, subsequently increasing the specific surface area available to interact with the analyte [145]. The maximum NO_2_ responses (Rg/Ra) with rGO/SnO_2_ and pristine SnO_2_ were 227.6 and 34, respectively, at a working temperature of 75 °C (Figure 7a).

Wang et al. (2019) also mentioned two primary reasons that enhanced the gas-sensing properties via rGO/SnO_2_ hybridization. Firstly, rGO had a large specific surface area, which aided in improving the surface accessibility. The introduced rGO sheets significantly suppressed SnO_2_ aggregation by covalently attaching SnO_2_ nanoparticles to their surfaces. The SnO_2_ nanoparticles were then kept apart from the rGO sheets to avoid graphitization. The nanocomposite was constructed with a large specific surface area (197.54 m^2^/g) to enhance the diffusion of gas molecules, as previously described. This resulted in an increase in the gas response and a more rapid response recovery time. Secondly, the electrical properties of the rGO were modulated due to the rGO’s *p*-type conductivity. In the rGO/SnO_2_ heterojunction structures, two distinct potential barriers and depletion layers coexisted comprising the SnO_2_ grain boundary and the interface between rGO and SnO_2_. As reported in the literature, the work function of rGO and SnO_2_ was approximately 5.4 eV [146] and 4.8 eV [147], respectively. Gas molecule adsorption or desorption altered both potential barriers, causing a change in the overall resistance of the sensor.

The energy band models for rGO/SnO_2_ hybridization are schematically presented in Figure 7b,c. The adsorption or desorption of gas molecules would change both the potential barriers that cause the modulation of the total sensor resistance. As illustrated in Figure 7b, when the sensor is exposed to air, the adsorbed oxygen molecules on the surface of rGO/SnO_2_ are ionized to form O_2_^−^, O^−^, or O^2−^ by absorbing free electrons from the materials’ surface, thereby affecting the sensor resistance. The released NO_2_ (Figure 7c) interacts with the adsorbed oxygen species and is chemisorbed as NO^2−^ ions, contributing to further electron extraction from the rGO/SnO_2_. This resulted in a higher potential barrier height and a more comprehensive depletion layer for both types of depletion layers. Therefore, electrons would have a much more difficult time traveling through the SnO_2_ nanoparticles, significantly increasing the sensor resistance. Benefitting from the SnO_2_ nanosize, the density of NO_2_ adsorption sites increased significantly, further widening the EDL and substantially increasing the sensor response and selectivity. The high electron mobility of rGO may also vastly accelerate the electron transport between the gas and the sensing layer, leading to a relatively swift response and recovery time at lower temperatures compared to all pristine metal oxides [143].

**Figure 8:**
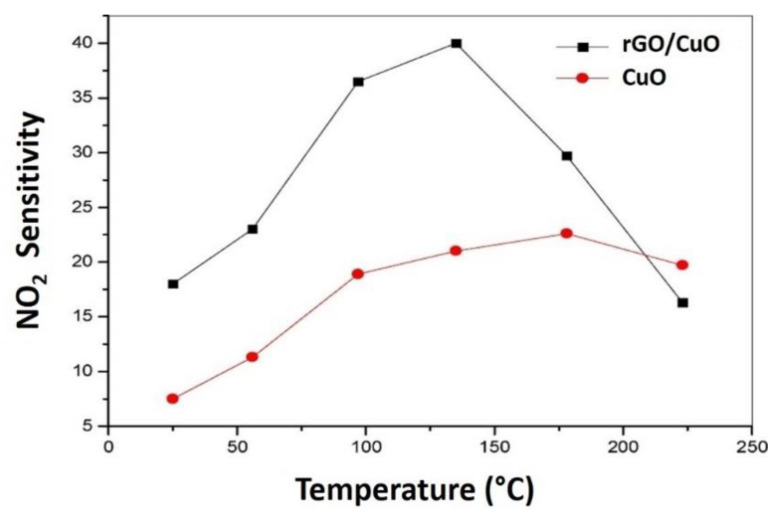
Comparison of the sensing performance of an rGO/CuO-based sensor with pristine CuO at 5 ppm NO_2_ with temperatures from 30 °C to 225 °C. Reproduced from ref. [148].

Previously, a similar rGO/CuO sensor was fabricated by Li and colleagues for NO_2_ detection [148]. The hybridization of rGO/CuO was synthesized at room temperature via a one-pot process and deposited onto a Si/SiO_2_ wafer. When exposed to 1 ppm NO_2_ at room temperature, the sensor demonstrated an excellent sensing performance with a highly sensitive response (14) as well as a response time and recovery time of 66 s and 34 s, respectively. At room temperature, the low detection limit was observed to be as low as 60 ppb. The higher sensing response was possibly due to the large surface area of the rGO/CuO nanohybrids, which facilitates the carrier transfer between NO_2_ molecules and the nanohybrids. Additionally, Li investigated the sensing performance of rGO/CuO nanohybrids and the performance was compared to that of pristine CuO in the presence of 5 ppm NO_2_ at various temperatures (Figure 8). The temperature was found to have a huge influence on the NO_2_ sensitivity. In particular, the rGO/CuO/rGO and pristine CuO gas sensors demonstrated an outstanding response performance as the temperature increased from 30 °C to 135 °C. However, once the temperature reached 135 °C, the sensitivity of the rGO/CuO sensor showed a decreasing trend, which may be associated with the controlled NO_2_ molecular absorption process on the surface of CuO even at elevated temperatures. The mechanism underlying the high NO_2_ selectivity can be explained in two ways. Firstly, when the nanohybrids are exposed to NO_2_ gas, the adsorbed NO_2_ molecules on the sheet-like CuO may have captured electrons from the *p*-type CuO to form NO_2_^−^. This essentially increased the hole concentration in the sheet-like CuO. Secondly, the large surface area of the rGO provided numerous adsorption sites for NO_2_ molecules, where the rGO could also act as a fast carrier transport channel due to its high mobility, which increased the carrier transfer to the collection electrodes. Besides, a large number of interfaces, grain boundaries, and surface defects in the nanohybrid structures benefitted the sensitivity of NO_2_ attraction [149].

### 7.2. Ammonia (NH_3_) Gas Detection

NH_3_ is a hazardous toxic gas and continuous exposure to the gas causes severe irritation and potential death to humans although it is a vital component in many fields, including industries and refrigeration systems [150]. NH_3_ leakage of more than 25 ppm is considered toxic to humans but the detection limit of NH_3_ by the human nose is only up to 50 ppm [151]. According to the Department of Occupational Safety and Health Malaysia (DOSH), NH_3_ has a distinctive pungent odor that can be identified by smell at concentrations as low as 5 ppm. However, the Permissible Exposure Limit (PEL) is 25 ppm or 17 mg/m^3^ for an eight-hour time-weighted average airborne concentration [24]. Therefore, the selective sensing of NH_3_ is essential in this regard to detect and treat environmental contamination and health issues.

**Figure 9:**
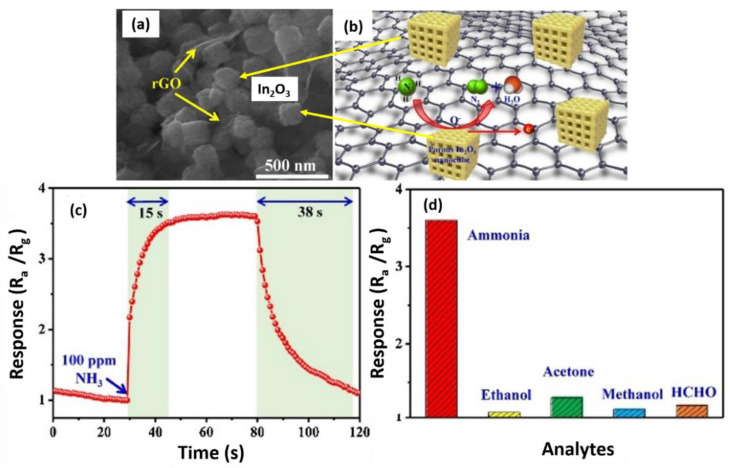
(**a**) SEM image of the rGO/In_2_O_3_ nanocomposite, (**b**) The schematic diagram of the rGO/In_2_O_3_ nanocomposite detecting the NH_3_, (**c**) room temperature response and recovery time of the rGO/In_2_O_3_ nanocomposite up to 100 ppm NH_3_, and (**d**) rGO/In_2_O_3_ sensor responses to 100 ppm of various gases at room temperature. Reproduced from ref. [154].

In one study, a novel hybrid rGO/In_2_O_3_ nanocube-based sensor (Figure 9a,b) was developed through a simple electrostatic self-assembly method for the detection of NH_3_ at room temperature [152]. The structural design and rational integration of the rGO sheets in the hybrid nanocomposite demonstrated high sensitivity, rapid response, and outstanding selectivity toward NH_3_. At 100 ppm NH_3_, the sensor response was 3.5, as illustrated in Figure 9c. Furthermore, the sensing capacity of the hybrid rGO/In_2_O_3_ recorded a swift response time of 15 s and a recovery time of 38 s. Note that the gas sensor response and recovery time are critical parameters for sensing applications, where they determine the time taken for the gas sensor to achieve 90% of its maximum response after being exposed to the target gas and the time taken to return to 10% of its stabilized value in the target gas after being placed with clean air, respectively [153]. Additionally, the concentration-dependent response curve of the hybrid rGO/In_2_O_3_ exposed to various NH_3_ concentrations revealed that the sensor response increased linearly as the concentration increased from 100 ppm to 1000 ppm. The selectivity of the hybrid rGO/In_2_O_3_ when exposed to five different gases at maximum concentrations of 100 ppm at room temperature also demonstrated exceptional sensitivity to NH_3_ but a feeble response to the other five gases (Figure 9d).

The porous nanostructures of In_2_O_3_ were considered the main factor that promoted the sensing ability of the rGO/In_2_O_3_ for gas diffusion and adsorption. The high specific surface area of the nanocomposite provided more active sites and promoted gas adsorption [154]. The hetero-interfaces between In_2_O_3_ nanocubes and rGO may also influence the sensing performance. By introducing a small amount of rGO into the samples, it was possible to sufficiently disperse the rGO and reduce the physical contact between adjacent rGO sheets. Subsequently, numerous large interfaces between the In_2_O_3_ nanocubes and rGO were formed, facilitating the transfer of electrons between them. Moreover, the *p*-*n* heterojunction provided additional active sites, such as point defects and vacancies that were critical for improving the sensor performance. As discussed previously, the presence of rGO may enhance the sensor properties via the formation of *p*-*n* heterojunctions and the porous structure of the metal oxide.

**Figure 10:**
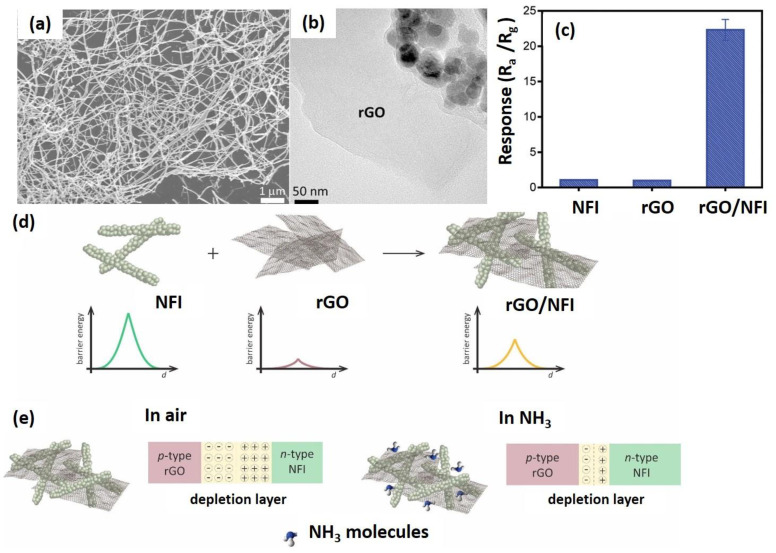
TEM image of (**a**) NFI, (**b**) rGO/NFI, (**c**) response of rGO/NFI exposure to 15 ppm NH_3_ at room temperature, (**d**) schematic diagram of the potential barrier energy, and (**e**) configuration of the *p*-*n* heterojunction EDL of the hybrid rGO/NFI in the air and NH_3_ atmosphere. Reproduced from ref. [155].

Other research has investigated the utilization of In_2_O_3_ ceramic nanofibers (NFI) (Figure 10a), an *n*-type semiconductor with an energy bandgap of 3.6 eV hybridized with rGO for NH_3_ detection at room temperature [155]. The interconnected mesoporous hierarchical structure of NFI was the crucial feature that allowed NH_3_ molecules to diffuse and reach the adsorption centers at the core of the nanofiber structure [156]. Since In_2_O_3_ is also known to exhibit a low conductivity at ambient temperatures due to its high potential barrier energy at the grain boundary [157], an alternative method to address this limitation was to generate a *p*-*n* heterojunction by combining the In_2_O_3_ (*n*-type) with a *p*-type semiconductor, which lowers the overall potential barrier energy and thus improves the conductivity [158]. The sensing performance of the hybrid rGO/NFI (Figure 10b) demonstrated several noticeable results, including a rapid response to 15 ppm NH_3_ with a sensitivity 10 times higher than that of the pristine NFI and rGO at ambient temperatures (Figure 10c), a low detection limit of 44 ppb, and an excellent selectivity for NH_3_ over other nitrogenated compounds and organic solvents. The potential barrier formed in metal oxides, such as In_2_O_3_, was sufficient to prevent the electron from flowing naturally through the interface layer (Figure 10d). In the presence of air, oxygen molecules are adsorbed onto the surface, capturing electrons from the In_2_O_3_ conduction band, and forming the EDL at the In_2_O_3_ boundary [159]. When exposed to the analyte, the gas molecules interact with the oxygen species and reduce the EDL. However, the interaction was insufficient to change the resistive response due to the high energy of the potential barrier [160].

For hybrid rGO/NFI nanocomposites, the *p*-*n* heterojunction generated at the interface reduced the potential barrier, permitting electrons to flow from the *n*-type In_2_O_3_ to the *p*-type rGO until the Fermi level reached equilibrium [161]. Once the rGO/NFI was exposed to NH_3_ in the air, the gas molecules interacted with the NFI’s adsorbed oxygen species and the rGO adsorption centers (Figure 10e). As a result, the electrons returned to the sensitive material and lowered the overall resistance. Therefore, the improved NH_3_ detection by the hybrid rGO/NFI can be attributed to three major factors: (1) the rGO-functionalized NFI increased the active sites for the adsorption of NH_3_ gas molecules, and (2) the synergistic effect between the NFI nanofibers and rGO sheets formed a 3D interconnected structure (*p*-*n* heterojunction formed between NFI and rGO surfaces) that facilitated the gas accessibility for more adsorption centers [162]. In short, the specific hybrid rGO/NFI is beneficial for enhanced and selective NH_3_ sensing, proving that the fabricated hybrid rGO/NFI nanocomposite has great potential for a sensor application with an outstanding performance.

Apart from that, Wang et al. (2017) fabricated the rGO/ZnO nanowire nanocomposite using the dip-dropping method to detect NH_3_ at room temperature [163]. The sensing performance of the device exhibited an outstanding response of 19.2% (at 25 wt.% of ZnO loading and rGO 75 wt.%) when exposed to a lower concentration of NH_3_ (50 ppm) at room temperature, which was higher than that of the pristine rGO-based sensor of only 3.05% [164] and pristine ZnO at 500 ppm NH_3_ with a response of 8% [165]. It was revealed that ZnO nanowires could act as electron transfer pathways on rGO sheets, which corresponded to the improved response, response time, recovery time, stability, and selectivity of the rGO/ZnO nanowire nanocomposite-based sensors. While ZnO is a typical *n*-type semiconductor, it occasionally exhibits *p*-type semiconductor characteristics due to various possible causes [166,167,168]. The possible causes demonstrated that: (1) trace amounts of nitrogen or carbon were doped into ZnO nanowires as a result of the presence of N_2_ and graphite during the carbothermal reduction process, and (2) ZnO nanowires and metal electrodes formed a Schottky barrier that affected the sensor to exhibit the *p*-type semiconductor performance. Furthermore, the Fermi level of ideal intrinsic graphene was close to the Dirac point, indicating that this was not a *p*-type nor *n*-type semiconductor behavior. Nevertheless, the possibility to adsorb other molecules in the air similar to water molecules was typically because of the presence of oxygen-containing groups from rGO samples, which resulted in the electron transfer from the rGO to oxygen-containing groups or adsorbed molecules. Thus, the hole became the primary carrier and exhibited *p*-type semiconductor characteristics.

**Figure 11:**
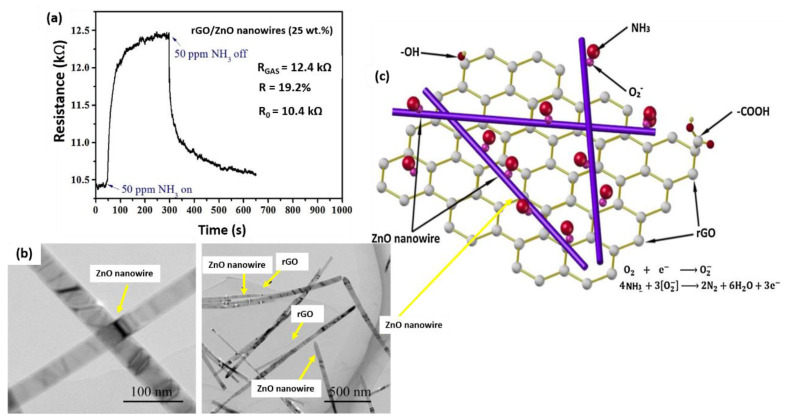
(**a**) The response curve of rGO/ZnO nanowires when exposed to 50 ppm NH_3_ at room temperature, (**b**) TEM image of rGO/ZnO nanowire nanocomposite, and (**c**) the sensing mechanism of rGO/ZnO nanowires towards NH_3_ detection. Reproduced from ref. [163].

As a reducing gas, NH_3_ contains a single electron pair that can easily be donated to the *p*-type rGO sheets, thereby increasing the resistance of rGO-based nanocomposites. The rGO, which acts as a base material, contributes significantly to the sensor’s response. However, a homogeneous heterojunction is formed when rGO is hybridized with ZnO nanowires, facilitating the electron transfer, and lowering the activation energy required for NH_3_ molecules to interact with the surface of the sensitive material. Thus, the sensor demonstrated an excellent NH_3_ detection performance when exposed to 50 ppm NH_3_ at room temperature (Figure 11a). The sensing mechanism is initiated by the adsorption behavior of NH_3_ molecules on the rGO/ZnO nanowire nanocomposite. Since rGO/ZnO nanowires exhibit a higher electrical conductivity than rGO-based nanocomposites, it was assumed that ZnO nanowires acted as a network and offered alternative pathways for electron transfer. As a result, the resistance of rGO was decreased and the resistance change at the rGO sheets was accelerated, which enhanced the sensing properties of the hybrid nanocomposite. The oxygen molecules were first ionized and then deposited on the rGO/ZnO nanowires nanocomposite to form the precursor layer. Following that, the target NH_3_ molecules were directly absorbed onto the precursor layer and reacted with O^2−^. Figure 11b illustrates the rGO/ZnO nanowire nanocomposite under TEM observations, while Figure 11c shows the sensing mechanism of the rGO/ZnO nanowires nanocomposite toward NH_3_.

### 7.3. Hydrogen (H_2_) Gas Detection

H_2_ has been proposed as a novel sustainable, efficient, and clean energy carrier among the numerous industrial gases. However, H_2_ accumulation might lead to catastrophic explosions [169]. Therefore, the development of portable and small-sized H_2_ sensors suited to the industrial field is crucial for future H_2_-related applications [170].

**Figure 12:**
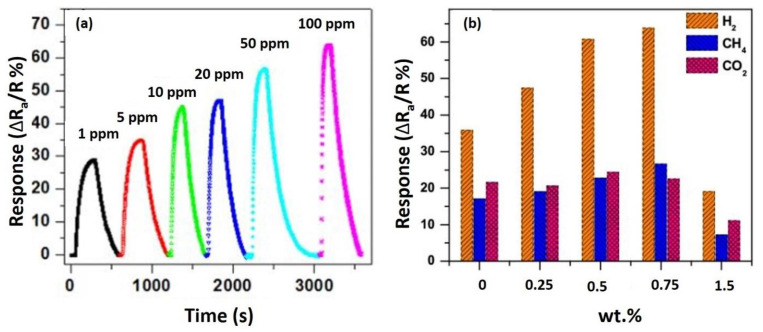
(**a**) Response of rGO-Ni-doped ZnO nanostructures toward H_2_ concentration of 1–100 ppm at 150 °C for 0.75 wt.% rGO loading and (**b**) the selectivity response for pristine rGO and various rGO loaded-Ni-doped ZnO nanostructure-based sensors. Reproduced from ref. [171].

An interesting study on the effect of rGO loading concentrations on the sensitivity and LOD of H_2_ was reported by Bhati et al. (2018) [171]. The sensor was developed using rGO-Ni-doped ZnO nanostructures and the RF sputtering was used to grow Ni-doped ZnO nanoplates. The Hummers’ method was employed to produce the rGO using the drop cast process at various rGO loading concentrations ranging from 0 wt.% to 1.5 wt.%. The use of 0.75 wt.% rGO-Ni-doped ZnO sensor for H_2_ detection demonstrated a maximum relative response of 63.8% to 100 ppm H_2_ at 150 °C (Figure 12a), as well as excellent selectivity for H_2_ over methane and CO_2_ (Figure 12b) even at lower gas concentrations (1–100 ppm). The increased relative response was largely due to the synergistic effect of the highest number of *p*-*n* heterojunctions with large Schottky barrier variations and more oxygen ions available for adsorption on rGO for H_2_ interaction. Yet, the lower response recorded using 1.5 wt.% rGO loading could be due to the established interconnected rGO between electrodes, which reduced the overall resistance and allowed the current to flow directly over the interconnected rGO. Consequently, these *p*-*n* heterojunctions containing 1.5 wt.% rGO (*p*-type) with Ni-doped ZnO (*n*-type) and the additional active sites caused by the Ni dopant were ineffective for H_2_ detection.

Previously, the palladium-tungsten trioxide (Pd-WO_3_) nanomaterial, was hybridized with rGO (rGO/Pd-WO_3_) for H_2_ detection [172]. The H_2_-sensing capability of the rGO/Pd-WO_3_ thin films was investigated at various H_2_ concentrations (20–10,000 ppm) and temperatures (25–250 °C). At 100 °C, the rGO/Pd-WO_3_-based sensor demonstrated an optimal sensitivity (tenfold than that of rGO/Pd-WO_3_) and a swift response and recovery time of less than 1 min. Additionally, the rGO/Pd-WO_3_-based sensor recorded a low sensitivity of H_2_ at room temperature of 20 ppm. The experimental findings proved the effect of residual oxygen-containing functional groups in rGO on the sensitivity of the GO/Pd-WO_3_-based sensor. The irregular and agglomerated structure of rGO/Pd-WO_3_ resulted in a decreased sensitivity of 14 and a shorter saturated time of 8 s, which was due to the limited number of active surface sites for hydrogen molecules to interact and the decreased porosity. Therefore, the incorporation of rGO rather than GO would increase the sensing performance of the Pd-WO_3_ nanostructures. Overall, the rGO/Pd-WO_3_-based sensor with hierarchical nanostructures provided efficient H_2_ -sensing active sites.

When combined effectively, these factors enhanced the sensitivity of the hybrid rGO/Pd-WO_3_ nanostructure by two orders of magnitude and increased the response time to 45 s. Based on the rapid response time of less than 1 min, increased sensitivity of 102, and improved recovery time over a wide range of gas concentrations, the optimal operating temperature was determined at 100 °C. Moreover, the sensor can be used at room temperature to improve the recovery time subjected to a brief heating interval. It is worth noting that the temperature around 100 °C was chosen as the best operating temperature range for metal oxide-based gas sensors hybridized with graphene due to the energy consumption, safety, and humidity concerns.

### 7.4. Hydrogen Sulfide (H_2_S) Gas Detection

H_2_S is a colorless noxious gas that is released from sewage sludge, sulfur-containing organic matter decomposition, and microbial sulfate reduction [173]. Although H_2_S emits a pungent odor reminiscent of “rotten eggs”, humans are unable to detect the dangerous concentration of H_2_S in time due to the factory hyposensitization. Inhalation of H_2_S has a detrimental effect on the nervous system and can result in unconsciousness [174]. Given the high risk associated with H_2_S, its detection is critical to protect humans and the industrial sector from unsuspected H_2_S leakages, particularly at room temperature.

Recently, a hybrid maghemite (γ-Fe_2_O_3_) octahedron with rGO (rGO/γ-Fe_2_O_3_)-based sensor for H_2_S detection was developed [175] by dispersing the γ-Fe_2_O_3_ octahedrons extracted from MIL-88 on the rGO interface. At room temperature, the ordered rGO/γ-Fe_2_O_3_ nanocomposite sensor exhibited an improved H_2_S detection with a sensitivity of 520 at 97 ppm H_2_S but lacked a broader response toward NH_3_, chloroform (CHCl_3_), NO, SO_2_, and formaldehyde (CH₂O) compared to pure γ-Fe_2_O_3_ sensors. The sensitivity mechanism of the rGO nanocomposite was contributed to by the larger accessible surface rGO, while the gas detection by γ-Fe_2_O_3_ was achieved based on the bulk resistance effect. Furthermore, the outstanding performance of rGO/γ-Fe_2_O_3_ toward H_2_S was contributed to by the excellent conductivity and abundance of active sites of the 2D rGO structure. The large surface area of the rGO nanocomposites facilitated gas diffusion, provided additional active sites for the reaction, and further enhanced the gas sensitivity. Additionally, the porous nature of Fe_2_O_3_ octahedrons facilitated gaseous molecule penetration into the rGO/γ-Fe_2_O_3_ nanocomposite.

Apart from that, the energy bandgap and electron affinity of Fe_2_O_3_ were 2.2 eV [176] and 4.7 eV, respectively [177], and the work function of rGO was approximately 4.8 eV [178], which was lower than that of γ-Fe_2_O_3_. The heterostructure configuration was formed through the hybridization of rGO/γ-Fe_2_O_3_ in which the transferred electrons generated more chemisorbed oxygen species on the surface of γ-Fe_2_O_3_, resulting in the formation of more active sites [179] and improved the H_2_S detection. The rGO with a high carrier mobility influenced the conductive network in the rGO/γ-Fe_2_O_3_ nanocomposite, which resulted in the rapid transfer of carriers. Moreover, the addition of γ-Fe_2_O_3_ octahedrons hindered the stacking of rGO nanosheets as well as provided more channels for diffusion to transport H_2_S molecules across the rGO layers. Therefore, these factors influenced the interface electrical properties of the rGO/γ-Fe_2_O_3_-based sensor, substantially enhancing the sensing performance toward the H_2_S at 97 ppm at room temperature with a high response of 520.

### 7.5. Carbon Dioxide (CO_2_) Gas Detection

CO_2_ is a colorless and odorless greenhouse gas found in nature. It performs an essential function in many living organisms on this planet although it is present in smaller percentages compared to other gases in the atmosphere. Nevertheless, CO_2_ monitoring is critical for managing air quality, particularly in the health and clinical sectors, as over-inhalation of CO_2_ by humans results in intoxication and hypercapnia or hypercarbia. In fact, a CO_2_ level of 2000–5000 ppm in an indoor room could lead to an increase in a person’s instant heartbeat and induce a state of unconsciousness [180].

The development of a highly selective, straightforward, and cost-effective CO_2_ gas sensor was reported using stabilized rGO/NiO-In_2_O_3_ nanosphere-sensing electrodes [181]. The CO_2_ gas-sensing electrode operates at room temperature via a simple hydrothermal method aided by sonication to enhance the decoration of rGO layers at different ratios (9:1, 8:2, 7:3, and 6:4) of NiO and In_2_O_3_ nanoparticles (for example, the composition of rGO-9:1 nanocomposite 0.9 M of NiCl_2_.6H_2_O and 0.1 M of InCl_3_.4H_2_O) using the intense reductant hydrazine hydrate. The results indicated that the developed sensor rGO-8:2 had a high sensitivity of 40% to CO_2_ at 50 ppm. Additionally, the sensor could detect CO_2_ concentrations up to 5 ppm with a 6 s response time and a 5 s recovery time. Besides, the optimal amount of In_2_O_3_ addition in combination with NiO had a significant effect on CO_2_ detection. Additionally, the fabricated electrode demonstrated a 95% long-term stability over 50 days when observed at 10-day intervals.

Another room temperature-operating CO_2_ detection sensor through the hybridization of rGO/SnO_2_ was developed by Lee et al. (2021) [182]. The synergistic effect of hybridization with the metallic conductivity of rGO and SnO_2_ increased the detection limit at room temperature and 58% relative humidity to sub-ppm (5 ppm) and excellent CO_2_ sensing. Moreover, the response change of the hybrid rGO/SnO_2_-based sensor to 100 ppm CO_2_ was 1.206%, which was 6.7 times greater than the response change of pristine rGO (0.179%). An excellent gas interaction with lower CO_2_ concentrations at room temperature resulted in a simple fabrication, low-cost production, and low power consumption that could be used to develop a portable CO_2_ gas sensor.

### 7.6. Sulfur Dioxide (SO_2_) Gas Detection

Sulfur dioxide (SO_2_) is a colorless gas composed of sulfur and oxygen, which is very harmful to humans. The gas is mainly produced from the combustion of coal, fuel, oil, and other sulfur-containing substances, making it one of the major pollution gases in the atmosphere [183]. Even at low concentrations, prolonged exposure can cause irritation of the skin and eyes, lung failure, sore throat, and, in severe cases, death [184]. Therefore, it is important to fabricate SO_2_ sensors with rapid and accurate detection, especially at room temperature sensitivity.

Previously, the in situ one-pot polyol method was employed to fabricate nanocomposite films of Multiwalled carbon nanotubes/Tungsten trioxide (MWCNTs/WO_3_) and rGO/WO_3_ on an alumina substrate for room temperature and ppb-level SO_2_ gas detection [185]. The gas-sensing result showed that the SO_2_ sensor with the rGO/WO_3_-based sensor film exhibited the highest response at 30% for very low SO_2_ concentrations from 50 ppb to 300 ppb at room temperature compared to that of the MWCNTs/WO_3_ nanocomposite film (around 25% of response at 300 ppb SO_2_) and the pristine WO_3_ film (around 10% of response). The rGO/WO_3_-based gas sensor also showed a fast response and recovery time, high reproducibility, and the lowest detection limit for SO_2_. The enhanced responsiveness was influenced by the formation of new conducting pathways and the spreading of the EDL at the interface of the doped MWCNTs and rGO with the WO_3_ nanoparticles toward the interaction with SO_2_ gas.

In comparison, MWCNT/SnO_2_ and rGO/SnO_2_ hybrid nanocomposites as SO_2_ gas sensors were fabricated by chemically hybridizing MWCNTs and rGO into a colloidal SnO_2_ nanoparticle solution, respectively [186]. At 220 °C and 500 ppm SO_2_, the pristine SnO_2_ sensor recorded a sensing response of 1.2, which was significantly improved to 22 for the rGO/SnO_2_-based sensor compared to the response of 5 for the MWCNT/SnO_2_-based sensor at a lower operating temperature of 60 °C and the same SO_2_ concentration of 500 ppm. The modification of the space charge region at the interface of the *n*-SnO_2_ and *p*-rGO in response to the target SO_2_ gas was synergized to enhance the SO_2_ detection response. Additionally, the response and recovery times for the hybrid rGO/SnO_2_-based sensors were slower at all temperatures compared to the pristine SnO_2_-based sensor. At 200 °C, the response and recovery times of pristine SnO_2_ thin-film sensors were 5.2 min and 7.4 min, respectively. In contrast, the response and recovery times for the rGO/SnO_2_ sensor at 60 °C were 2.4 min and 3.5 min, respectively, while the response and recovery times for the MWCNT/SnO_2_ sensor were 5.3 min and 4.3 min, respectively. This could be due to the limited formation of the conducting channels by rGO films and MWCNTs between the electrodes, allowing a higher charge transfer rate, and thus a faster response and recovery times for the hybrid nanocomposite sensors to detect SO_2_ at low temperatures. Table 5 tabulates the summary of the latest advancements in rGO/metal oxide-based gas sensors for NO_2_, NH_3_, H_2_, H_2_S, CO_2_, and SO_2_.

## 8. Conclusions and Future Perspective

This review has presented the advanced development of hybridized rGO/metal oxide nanocomposites as gas-sensing materials, which have significantly expanded the possibility for innovative and effective gas sensor devices. The hybridization of rGO/metal oxide has not only provided a considerable improvement in terms of the sensitivity and selectivity, but it has also allowed gas sensors to operate preferably at room temperature and below 200 °C for the detection of toxic gases. Nevertheless, the accomplishments described in this article are still within the realm of basic research, thus necessitating a considerable effort to transform lab-scale devices into reliable and robust practical applications, whereby the developed gas sensors are susceptible and exposed to unpredictable environmental conditions. Despite the reported findings from various studies that the performance of these sensors can be sustained for more than a half year, such stability and environmental resistance are not attainable in the real world. The ability to detect nonpolar and bulky molecules that are highly detrimental to safety, such as volatile organic compounds, is still under investigation. These molecules react differently with rGO/metal oxide sensors than polar molecules, while pristine graphene exhibited a poor surface affinity for them, making the detection more challenging. Regardless of the drawbacks, the ease of processing and fabrication, doping capability, small device configurations, compatibility with various substrates, and high-temperature tolerance are some of the favorable performances of rGO/metal oxide-based gas sensors due to their high sensitivity and selectivity to gases at temperatures as low as 30 to 50 °C. This implies a potentially huge opportunity for their practical use as gas sensors.

The main limitation to the practical implementation of rGO/metal oxide-based gas sensor includes the lack of reproducible, low-quality thin films, non-uniformity of film thickness in bi-layer/tri-layer rGO, high sheet resistance for layers’ tens of hundreds of layers thick in nm, relative inertness to the environment from the absence of active functional groups, and sensitivity to humidity. In real-life applications, the performance and capability of the gas sensors would be highly dependent on the relative humidity of the surroundings. Additionally, the primary bottleneck for the future development of gas sensing-devices is that the current large-scale application of rGO remains limited and at the same time the responsiveness and sensitivity of rGO as a gas sensor to specific gases is also limited, which should be enhanced through further treatment or surface modifications. According to the current development trend, the following factors may contribute to the improvement of response times: (1) increasing the specific surface area of rGO by coating it with functional molecules, such as boron or nitrogen; (2) hybridizing rGO with specific nanomaterials; (3) the lower energy bandgap of rGO and metal oxides themselves contributed to their use as effective gas-sensing materials; and (4) the success of electron transfer was aided via the efficacious design of sensing deviators.

Future research should focus on improving the shortcomings of rGO-based gas sensors, including: (1) developing an easy and economical synthetization of single and multi-layer rGO that are highly in demand; (2) minimizing the substrate effects to prevent undesirable surface contamination during the micro-fabrication processes; (3) improving the adsorbate sensitivity and selectivity novel by formulating functional molecules with specific interaction with rGO; and (4) measuring and monitoring the impact of humidity on the sensitivity of rGO-based gas sensors.

Moreover, the room temperature operation, sensitivity, and selectivity of the metal graphene/metal oxide hybrid sensors are needed for specific molecules to fulfil the demand for highly selective gas sensors. Apart from the hybridization process, surface functionalization with specific molecules/compounds on graphene also provides high selectivity to the target molecules with improved sensitivity. There are also mentions of some other forms of graphene through graphene nanoribbons and 3D graphene, which can significantly promote the charge transport in graphene while interacting with the foreign molecules showing their potential in sensing. The existent functional groups not only allow for good coordination towards molecules but also facilitate surface modification to enhance detection sensitivity further. With further exploration, it is expected that the hybridization of rGO with metal oxide-based gas sensors would possibly lead to its extensive applicability as an ambient gas sensor with an accelerated response/recovery speed in the future.

The most concerning aspect of metal oxide-based sensor operation for toxic gas sensors is that the high operating temperature cannot be stopped by reducing the operating temperature. However, humidity plays a crucial role in the gas sensor, and the adsorption of H_2_O molecules will affect the adsorption and reaction of the target gas on the sensor surface, thereby reducing the sensitivity of the gas sensor. In the future, more attention could be paid to improving the insensitivity to humidity for preparing a toxic gas sensor with a high performance and high humidity resistance suitable for practical applications, especially in high humidity areas in the jungle and equator line areas such as Malaysia and Indonesia. It is optimistic that such advanced improvement of rGO-based sensitive materials would play a significant role as a gas-sensitive material in the future with more incredible benefits in various research.

## Figures and Tables

**Table 1:** Performance comparison between the graphene-based gas sensor and other types of gas sensors.

Sensor Type	Performance and Capacity
Graphene-based gas sensor	- The gas-sensing mechanism is based on the adsorption/desorption of the two-dimensional (2D) atom-thick gaseous molecules on the graphene surface, which leads to altered properties - Sensors detect a voltage change and the resistance received in the presence of an analyte - Micrometer-sized graphene sensors are capable of detecting single gas molecules attached to or detached from the graphene surface by their large surface area, electrical conductivity, high electron transfer rate, and capacity to immobilize different molecules
Polymer-based gas sensor	- Polymer-absorption sensors are the most common type of gas sensor that can measure the change in resistance of an electrically active sensitive material upon exposure to a target gas analyte - The presence of the π-electron conjugation system (with *p*-type conductive behaviors) along the polymer chain, which allows the formation of delocalized electronic states, results in a resonance-stabilized structure of the polymer - The electrical conductivity can be changed upon exposure to oxidative or reductive gas molecules at room temperature. However, they exhibit a low sensitivity, poor stability, and gas selectivity due to their relatively low conductivity and high affinity toward volatile organic compounds and water molecules, which hinder their practical application - They behave as either an electron donor or electron acceptor when interacting with gas molecules, which can increase or decrease the carrier concentration, subsequently affecting the electrical conductivity or resistance of the sensing polymers
Chemiresistors gas sensor	- Metal oxide gas sensors are also known as chemiresistor gas sensors - The detection principle is based on the change in the resistance of a thin film upon the adsorption of gas molecules on the surface of a semiconductor - The gas–solid interaction affects the resistance of the film due to the density of the electronic species in the film - The target gas is identified using the change in electrical resistance, which translates to the gas concentration - Sensitive to environmental factors with a high energy consumption
Optical-based gas sensor	- Infrared (IR)-source sensors are widely used in optical-based gas sensors to provide a straightforward system with a high sensitivity, selectivity, and stability compared to non-optical methods with a much longer lifetime - The gas sensor operates when the IR source emits broadband radiation, including the wavelength absorbed by the target gas. The sample gas in the gas cell absorbs the radiation in a specific mechanism. Then, an optical filter is used to block all radiation except for the wavelength that is absorbed by the target gas. Finally, the presence of the target gas can be detected and measured by an IR detector - Simple operation procedures without oxygen and unaffected by electromagnetic interference but may be affected by ambient light interference
Calorimetric-based gas sensor	- The principle of calorimetric gas sensors is based on the change in temperature at catalytically active metal surfaces, such as platinum, palladium, or rhodium - The target gas is burnt to generate a specific combustion enthalpy, enabling the detection of low concentration analytes in a short response time - The Limit of Detection (LOD) for calorimetric sensors is typically in the low parts per thousand (ppt) range, which is suitable for industrial settings but insufficient for laboratory applications
Electrochemical-based gas sensor	- Electrochemical-based gas sensors measure the concentration of a specific gas within an external circuit from the resulting current due to the oxidization or reduction of the target gas at an electrode - The sensitivity and selectivity toward the target gas are crucial factors to obtain effective detection. Hence, the use of surface-modified electrodes with immobilized recognition gases elements is an effective technique to achieve the high selective and sensitive binding of the target compounds and real-time measurements
Capacitance-based gas sensor	- The system measures the dielectric constant of conductive films between the electrodes as a function of the gas concentration to express the electric flux, which is the equivalent of relative magnetic permeability - The capacitive sensor depends on Interdigitated Electrode (IDE) structures, which correspond to the two standard capacitor plates to monitor the dielectric coefficient changes of the film - Basically, a film with a lower dielectric constant than that of the analyte would increase the capacitance and vice versa
Acoustic wave-based gas sensor	- Sound-based gas sensors are also known as Surface Acoustic Wave (SAW)-based gas sensors - Any changes to the characteristics of the propagation path of an acoustic wave on the surface of the material would affect the wave velocity and/or amplitude - The frequency or phase characteristics of the sensor measures the changes in velocity that correspond to the measured physical quantity - An acoustic wave sensor contains a receptor, which is a component that is sensitive to an analyte and a transducer-like element that converts the response into an electrical signal

**Table 2:** The characteristics of graphene, GO, and rGO as gas sensors and their remarks.

Characteristics	Graphene	GO	rGO
Carbon (C) and oxygen (O) composition (%)	C (99) O (-)	C (62–65) O (35–48)	C (77–87) O (13–22)
Number of layers	3–5	1–3	1–3
Layer thickness (nm)	0.34	0.76–0.84	0.35–0.36
Electrical conductivity (S/m)	~1000	Non-conductive	~667
Remark as gas-sensing material	The absence of a bandgap or functional groups in pristine graphene limits its use in gas-sensor applications	Contains attached alkoxy (C-O-C), hydroxyl (-OH), carboxyl (-COOH), carbonyl (C=O), and other oxygen-based functional groups that provide GO with a very high resistance as a gas sensor. GO is suitable as a substrate for catalysis owing to its rich functionality, whereby chemical moieties over the surface of GO can be chemically altered	The presence of oxygen functional groups, vacancies, and defects, and sp^2^ bonded carbon, mean that rGO is a better choice for a gas-sensing application, especially when hybridized with a metal oxide to form *p*-*n* or *p-p* heterojunctions, allowing better toxic gas detection. rGO are more suited for certain applications, such as supercapacitors and batteries, due to their better electron transport properties
Advantages	High electrical and thermal conductivity, and high functionalization control by the sp^2^ hybridization structure	Water dispersibility, polar functionalization, and ease of processing due to the attached hydroxyl structure. GO structures also show hydrophilic behavior, while rGO shows hydrophobic behavior due to the loss of oxygen-containing compounds. In addition, the hydrophilic/hydrophobic behavior results in different dispersibility. GO shows a high dispersibility in aqueous media while rGO shows a significantly lower dispersibility	High electrical and thermal conductivity, and better functionalization control to adjust the desired physical and chemical properties of rGO according to the application
Disadvantages	Highly hydrophobic, high cost, and requires further functionalization for gas-sensing applications	Highly hydrophilic and low electrical and thermal conductivities. The combination of structural defects, poor dispersion, restacking, and multilayer thickness affects the electrical properties and high surface area of GO materials. The insulating nature of regular GO also limits its application in electronic devices and energy storage	Hydrophobicity properties related to the process used. The rGO bandgap varies from ~1.00 to 1.69 eV depending on the degree of reduction. The percentage of oxygen-containing functional groups in rGO is reduced and the percentage of sp^2^ carbon atoms is increased, which increase sits conductivity and makes it less electron-conducting (high ion conduction)

**Table 3:** Characteristics between *n*-type and *p*-type metal oxides toward the analyte.

Sensing and Responding Behavior	Example of Analyte	*p*-Type Metal Oxide Sensor	*n*-Type Metal Oxide Sensor
Reducing analyte	CO, NH_3_, C_2_H_5_OH	Increased resistance	Decreased resistance
Oxidizing analyte	NO, NO_2_, O_3_	Decreased resistance	Increased resistance
Dominant charge carrier	-	Holes (h^+^)	Electrons (e^−^)
Type of metal oxide	-	CuO, NiO, Cr_2_O_3_, Co_3_O_4_	ZnO, Fe_2_O_3_, SnO_2_, In_2_O_3_, WO_3_, TiO_2_
Response (S)	-	R_a_/R_g_ (Oxidizing) R_g_/R_a_ (Reducing)	R_g_/R_a_ (Oxidizing) R_a_/R_g_ (Reducing)

**Table 4:** Type of chemical sensors with their transduction mechanism.

Type of Sensor	Example of Sensor	Transduction Mechanism	Ref.
Electrical	Conductometric, capacitive, chemiresistive	The electrical transducer converts the mechanical energy into an electric signal, which may be voltage, current, or frequency.	[128,129]
Optical	Fluorescence, chemiluminescence, bioluminescence, surface plasmon scattering, evanescent waves interferometry	Quantify various properties of light, such as absorbance, photoluminescence, fluorescence, refractive index, optothermal effect frequency, wavelength, and polarization. These sensors rely on light detectors that convert light into electrical signals	[130,131]
Electrochemical	Potentiometric, amperometric ion-sensitive FET (ISFET), chemical FET (ChemFET)	An electrical current passes through a sensing electrode produced by an electrochemical reaction, which takes place at the surface of a sensing electrode coated with a catalyst, such as platinum	[132,133]
Electromagnetic	Hall sensors, Giant Magnetoresistance (GMR) sensors, Anisotropic Magnetoresistance (AMR) sensors, Magnetoimpedance (MI)	The measurand is converted to a voltage induced in the conductor via a change in the magnetic flux and in the absence of excitation. The electromagnetic transducer self-generating active transducers by the motion between a piece of magnet and an electromagnet is responsible for the change in flux	[134,135,136]
Piezoresistive	Capacitive pressure sensor, piezoelectric pressure sensor, Microelectromechanical Systems (MEMS) pressure sensor, optical pressure sensors	The basic principle of the piezoresistive pressure sensor is based on the use of a strain gauge made from a conductive material that changes its electrical resistance when it is stretched. For strain sensors under tension, the interconnected conducting network generates micro-cracks, which are the main source for the resistance change.	[137,138,139]
Piezoelectric	Quartz Crystal Microbalance (QCM), Surface Acoustic Wave (SAW)	A piezoelectric sensor function is when a physical dimension is transformed into a force and acts on two opposing faces of the sensing element. The detection of pressure variations in the form of sound is the most common sensor application, which is seen in piezoelectric microphones and piezoelectric pickups for electrically amplified guitars.	[138,140]
Thermal	Calorimetry	The transduction mechanism is initiated by the thermal effect generated by the specific chemical reaction or adsorption process between the analyte and receptor surface, which generates positive and negative charges	[141]

**Table 5:** Summary of the latest advances in rGO/metal oxide-based gas sensors for detection of the selected toxic gases.

Sensing Material	Target Gas Molecule	Gas Concentration (ppm)	Operating Temperature (°C)	Response	Response Time (s)	Recovery Time (s)	Ref.	Significant Remarks on the Selected Studies for the Heterojunction Effect between Metals and rGO for the Improvement on the Sensing Performance
SnO_2_/rGO	NO_2_	100	55	6.5%	-	500	[75]	Overall, it can be seen that pristine SnO_2_ and rGO have a very high resistance compared to both SnO_2_/rGO-4 (metal salt, Sn^4+^) and SnO_2_/rGO-2 (metal salt, Sn^2+^) nanocomposites exhibit a lowered resistance, and presented enhanced electronic conductivity, which could be ascribed to the formed *p*-*n* heterojunctions between SnO_2_ nanoparticles and rGO The formation of *p*-*n* junctions between SnO_2_ and rGO at the interface makes electron transfer easier, thus lowering the working temperature Oxygen vacancies and antisite defects from the SnO_2_/rGO porous nanocomposite act as channel entrances for the gas molecules and can effectively control the diffusion of small molecular gases. Therefore, it is difficult for large gas molecules to enter and diffuse in the 3D porous nanocomposite, resulting in fewer chances to react with the anion oxygen in the nanocomposite and leading to a sensing selectivity for particular gas molecules Theoretically, relatively higher temperatures can provide more energy to accelerate the transfer of electrons between the target gas and sensing materials, including the electrons in the interior of the nanocomposite, and can overcome the potential barrier between the SnO_2_ and rGO heterostructure
ZnO/rGO		100	RT	17.4%	780	1980	[187]	In this nanocomposite, as the concentration of the gas increases, the resistance decreases due to the NO_2_ adsorption and desorption process within the material that increases the response of the gas detection The rGO creates a conductive matrix that provides rapid electron channels to the hollow spheres of ZnO nanorods to assist in the sensing process. The Urc-ZGO nanocomposite material has a large specific surface area that allows sufficient contact area for the gas, ensuring that the NO_2_ molecules can easily penetrate and a high response for the sensing capacity
SnS_2_/rGO		11.9	80	56.8%	360	3180	[188]	
MoS_2_/rGO		3	160	1.23%	8	20	[189]	
ZnO/rGO		5	RT	25.6%	165	499	[190]	
ZnO/SnO_2_/rGO		5	RT	141.0%	33	92	[191]	
SnO_2_/rGO		5	RT	34.8%	70	39		
ZnO/rGO		5	RT	43.4%	272	1297		
In_2_O_3_ nanofibers/rGO	NH_3_	15	RT	23.37%	17	214	[155]	In_2_O_3_ (*n*-type) is combined with rGO (*p*-type) and the charge transfer is greatly enhanced which causes a reduction in the resistance once the presence of rGO increases the charge carriers’ mobility In_2_O_3_/rGO with a response 10 times greater than pristine In_2_O_3_ and rGO. The better performance of NFI-rGO can be related to the formation of the *p*-*n* heterojunction and the synergistic effect between the In_2_O_3_ nanofibrous structure and the rGO sheets, which features an increased relative response modulation For metal oxides such as In_2_O_3_, the potential barrier formed is high enough to prevent the electron from flowing naturally through the interface. In this case, in presence of air, oxygen molecules are adsorbed on the surface capturing electrons from the In_2_O_3_ conduction band and forming the depletion layer at the In_2_O_3_ boundary While exposed to the analyte, the gas molecules interact with the oxygen species reducing the depletion layer. However, this interaction is not enough to change the resistive response due to the high energy of the potential barrier from the In_2_O_3_. The hybridization of In_2_O_3_/rGO nanocomposite and the *p*-*n* heterojunction formed at the interface decreases the potential barrier allowing the effectiveness of electrons to flow from the *n*-type In_2_O_3_ to the *p*-type rGO until the Fermi level reaches equilibrium. When In_2_O_3_/rGO is exposed to reducing gas such as NH_3_, the gas molecules interact with the adsorbed oxygen species available from the In_2_O_3_ and with the rGO adsorption centers The improved NH_3_ sensing performance by the hybridization of In_2_O_3_/rGO can be attributed to the synergistic effect between the NFI nanofibrous structure and the rGO sheets forming a 3D interconnected structure which can facilitate the accessibility of the gas to more adsorption centers
ZnO nanowires/rGO		50	RT	19.2%	50	250	[163]	
SnO_2_-nanorods/rGO		200	RT	1.3	8	13	[192]	
SnO_2_ nanoflakes/rGO		50	RT (15–45)	1.16	<60	<60	[193]	
Co_3_O_4_/rGO		20	RT	1.78	351	1199	[194]	
Co_3_O_4_ nanorods		500	160	2.3	-	-	[195]	
Cu_2_O/rGO		100	RT	1.75	28	206	[196]	
In_2_O_3_ nanocubes/rGO		100	RT	3.5	15	38	[152]	
TiO_2_ film/Pd/rGO		10	RT	15	184	81	[197]	
ZnO wires/rGO		0.5	RT	56	6	36	[198]	
TiO_2_/graphene	H_2_	0.5%	75	23%	33	~92	[199]	
		0.5%	100	30%	30	~67.7		
		0.5%	125	16%	16	61		
		0.5%	150	12%	17.5	~22.5		
WO_3_/graphene		0.1 vol.%	RT	-	<13	<43	[200]	
Pd/GQDs/WO_3_		3600	120	500	12	35	[201]	
Pd-WO_3_/GO		100	100	72	35	37	[172]	
SnO_2_ nanowires/GO		100	50	24	-	-	[202]	
SnO_2_/rGO	H_2_S	50	RT	33	2	292	[203]	
ZnO/rGO		2	RT	30	2400	1800	[204]	
α-Fe_2_O_3_ nanofibers/rGO		0.1	350	1.5	-	-	[205]	
		1	350	9.2	-	-		
Cu_2_O/rGO		1	40	20%	~250	-	[206]	
WO_3_/rGO		10	330	45%	7	55	[207]	The response of a metal oxide sensor is related to the working temperature. For example, the responses of the sensors to 40 ppm H_2_S at temperatures 75 °C to 375 °C showed the highest response at 330 °C, so 330 °C was chosen as the best working temperature for the H_2_S sensor The possible reasons for the improved responses of rGO/WO_3_ nanocomposites for 40 ppm H_2_S rGO/WO_3_ nanocomposites increased with the increase in rGO from 1.6 wt.% to 5.7 wt.%, which could provide more active sites for the adsorption of H_2_S molecules, improving the responses of the sensor The hybridization of rGO facilitated electron charge carrier transport through the rGO/h-WO_3_ nanocomposites, so the responses of the rGO/WO_3_ nanocomposites sensors were better than pristine WO_3_ Nevertheless, when the amount of rGO increased to 5.7 wt.%, the amount of rGO exceeded the percolation threshold, so the resistance changes of sensing materials were not obvious when exposed to H_2_S after the rGO loading was 7.2 wt.%. Additionally, the 3D hybrid nanostructure in S2 provided more conducting networks for charge transfer and more channels for gas diffusion
γ-Fe_2_O_3_/rGO		100	RT	520.73	~30	-	[175]	
NiCo_2_O_4_/rGO		100	RT	3.51	2	449	[208]	
NiO-In_2_O_3_/rGO	CO_2_	50	RT	40%	6	18	[181]	
Sb_2_O_3_/graphene		50	RT	~0.2	16	22	[209]	
Al_2_O_3_ graphene		100	RT	10.84	14	22	[210]	
TiO_2_/rGO	SO_2_	5	RT	11.14%	-	-	[211]	
SnO_2_/rGO		500	60	22	144	210	[186]	

RT = Room temperature; GQDs = Graphene quantum dots.

## Data Availability

Not applicable.
